# Homeostasis of phospholipids — The level of phosphatidylethanolamine tightly adapts to changes in ethanolamine plasmalogens

**DOI:** 10.1016/j.bbalip.2014.11.005

**Published:** 2015-02

**Authors:** Fabian Dorninger, Alexander Brodde, Nancy E. Braverman, Ann B. Moser, Wilhelm W. Just, Sonja Forss-Petter, Britta Brügger, Johannes Berger

**Affiliations:** a Department of Pathobiology of the Nervous System, Center for Brain Research, Medical University of Vienna, Spitalgasse 4, 1090 Vienna, Austria; b Heidelberg University Biochemistry Center, Im Neuenheimer Feld 328, 69120 Heidelberg, Germany; c Department of Human Genetics and Pediatrics, McGill University-Montreal Children's Hospital, 4060 Ste-Catherine West, PT-406.2, Montreal, QC H3Z 2Z3, Canada; d Peroxisomal Diseases Laboratory, The Hugo W Moser Research Institute, The Kennedy Krieger Institute, 707 N. Broadway, Baltimore, MD 21205, USA

**Keywords:** AA, arachidonic acid, AGPS, alkylglycerone phosphate synthase, BA, batyl alcohol, CDP, cytidine diphosphate, DHA, docosahexaenoic acid, GNPAT, glyceronephosphate acyltransferase, HDG, hexadecylglycerol, PC, phosphatidylcholine, PE, phosphatidylethanolamine, PlsEtn, ethanolamine plasmalogen, PS, phosphatidylserine, PUFA, polyunsaturated fatty acid, RCDP, rhizomelic chondrodysplasia punctata, SM, sphingomyelin, Plasmalogen, Compensation, Docosahexaenoic acid, Arachidonic acid, Alzheimer's disease, Peroxisome

## Abstract

Ethanolamine plasmalogens constitute a group of ether glycerophospholipids that, due to their unique biophysical and biochemical properties, are essential components of mammalian cellular membranes. Their importance is emphasized by the consequences of defects in plasmalogen biosynthesis, which in humans cause the fatal disease rhizomelic chondrodysplasia punctata (RCDP). In the present lipidomic study, we used fibroblasts derived from RCDP patients, as well as brain tissue from plasmalogen-deficient mice, to examine the compensatory mechanisms of lipid homeostasis in response to plasmalogen deficiency. Our results show that phosphatidylethanolamine (PE), a diacyl glycerophospholipid, which like ethanolamine plasmalogens carries the head group ethanolamine, is the main player in the adaptation to plasmalogen insufficiency. PE levels were tightly adjusted to the amount of ethanolamine plasmalogens so that their combined levels were kept constant. Similarly, the total amount of polyunsaturated fatty acids (PUFAs) in ethanolamine phospholipids was maintained upon plasmalogen deficiency. However, we found an increased incorporation of arachidonic acid at the expense of docosahexaenoic acid in the PE fraction of plasmalogen-deficient tissues. These data show that under conditions of reduced plasmalogen levels, the amount of total ethanolamine phospholipids is precisely maintained by a rise in PE. At the same time, a shift in the ratio between ω-6 and ω-3 PUFAs occurs, which might have unfavorable, long-term biological consequences. Therefore, our findings are not only of interest for RCDP but may have more widespread implications also for other disease conditions, as for example Alzheimer's disease, that have been associated with a decline in plasmalogens.

## Highlights

• PE accurately compensates for the lack of plasmalogens *in vitro* and *in vivo*.
• PE levels decrease to adapt to excess of ethanolamine plasmalogens (PlsEtn).
• Plasmalogen deficiency favors incorporation of arachidonic acid into PE.
• Docosahexaenoic acid in ethanolamine phospholipids decreases upon PlsEtn depletion.

## 1 Introduction

The lipid composition of biological membranes is of great importance for cellular functions. Its dynamic adaptation to specific requirements is a crucial feature of lipid membranes in mammalian tissues. The most abundant membrane lipids are phospholipids, which are typically amphipathic and, thereby, make up the characteristic lipid bilayer structure of biological membranes. Among the phospholipids forming the bilayer, the major classes are the glycerophospholipids phosphatidylcholine (PC), phosphatidylethanolamine (PE), phosphatidylserine (PS) and the ether phospholipids (including plasmalogens), as well as sphingomyelin (SM), a sphingolipid. Each of these is further subdivided into a variety of distinct species based on their fatty acid chains. Eukaryotic cells are able to fine-tune the mixture of these lipids according to their biological task [1]. Furthermore, membranes adapt to various environmental conditions, like for example circadian rhythms or changes in temperature, by altering their lipid composition [2,3]. The dynamic aspect of lipid membranes is additionally underscored by the existence of membrane rafts (formerly termed lipid rafts), small protein–lipid domains enriched in sphingolipids and sterols, two other prevalent membrane lipid classes in addition to glycerophospholipids. Membrane rafts assemble transiently and compartmentalize important biological functions like signal transduction [4].

**Fig. 1:**
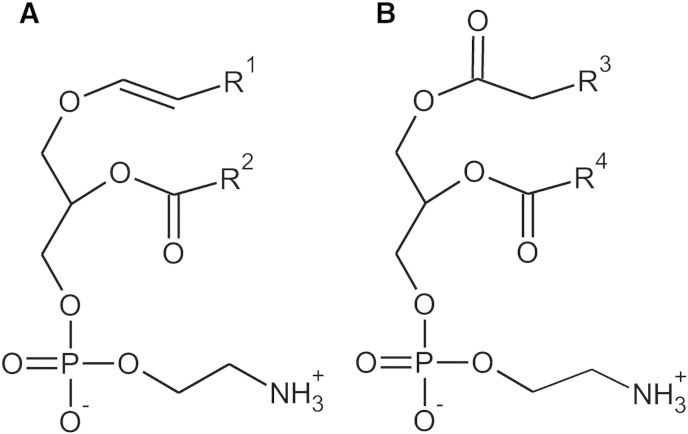
Structures of PlsEtn (A) and PE (B). R^1^ indicates the alkyl residues originating from the primary alcohols C16:0, C18:1 or C18:0. R^2^, R^3^ and R^4^ can be various fatty acyl residues.

Ether phospholipids constitute a special class of glycerophospholipids that have an O-alkyl group at their *sn*-1 position, which distinguishes them from the diacyl phospholipids. The most abundant subclass of ether phospholipids are the plasmalogens, which account for almost 20% of the phospholipid mass in humans [5]. These compounds carry a double bond adjacent to the ether bond, together forming a vinyl ether bond, which is characteristic of plasmalogens (Fig. 1A). Other ether phospholipids include plasmanyl phospholipids (containing a saturated ether moiety at the *sn*-1 position), platelet-activating factor, seminolipid, and, partly, the glycosylphosphatidylinositol (GPI) anchor of membrane proteins. The biosynthesis of ether phospholipids requires peroxisomes. In the lumen of these organelles, the concerted action of the two enzymes glyceronephosphate acyltransferase (GNPAT; alternative name: dihydroxyacetone phosphate acyltransferase (DHAPAT, DAPAT)) and alkylglycerone phosphate synthase (AGPS) generates the characteristic ether bond. The remaining biosynthetic steps, including the introduction of the vinyl double bond in case of plasmalogens, are subsequently accomplished at the endoplasmic reticulum (ER). The generation of the alkyl group at *sn*-1 depends on fatty alcohols that are either derived from dietary intake or synthesized by the fatty acyl-CoA reductases FAR1 or FAR2 [6]. Recently, FAR1, which preferably accepts saturated or monounsaturated C16 or C18 acyl-CoA esters as substrates, was suggested as the main reductase involved in plasmalogen biosynthesis [7]. Accordingly, C16:0, C18:0 and C18:1 are the major fatty alcohol species at the *sn*-1 position of plasmalogens. The fatty acid composition at *sn*-2 strongly depends on the cell type. In general, plasmalogens are enriched in polyunsaturated fatty acids (PUFAs), a fact that is especially pronounced in neurons. In brain white matter, however, monounsaturated species prevail to ensure myelin stability [8]. In most tissues, ethanolamine is the dominating head group. Choline plasmalogens play an important role in cardiac tissue, but represent a minor species in most other organs. Other head groups, like serine or inositol, are extremely rare. As major constituents of cellular membranes, plasmalogens shape membrane structure and dynamics. They also have been shown to be enriched in membrane rafts [9]. Furthermore, in some cell types, the frequent occurrence of PUFAs in the side chains of plasmalogens engages them as a storage depot for these essential fatty acids [10,11]. Additional functions like anti-oxidative action [12,13], stimulation of invariant natural killer T cells [14], membrane fusion [15,16] or constriction [17] and in generating lipid second messengers [18] have been proposed based on in vitro findings and experiments in ether phospholipid-deficient mouse models [19–22]. However, the exact biological roles of these lipids and the underlying molecular mechanisms are still enigmatic.

In humans, deficiency of ether phospholipids evokes rhizomelic chondrodysplasia punctata (RCDP), a rare, autosomal recessive disorder caused by mutations in the genes encoding the peroxisomal enzymes GNPAT (RCDP type 2) and AGPS (RCDP type 3) or peroxin 7 (PEX7; RCDP type 1), the receptor needed for peroxisomal import of AGPS [23–27]. Affected individuals suffer from a variety of severe symptoms, including growth and mental retardation, shortening of the proximal long bones, epiphyseal stippling, cataracts and joint contractures. In its severest form, RCDP is fatal during the first months of life, predominantly due to respiratory failure [28]. However, also less severe variants (“intermediate phenotype”) of the disease have been described, where residual plasmalogen biosynthesis slightly alleviates the symptoms [29–34]. Ether phospholipid deficiency is also a key feature of peroxisome biogenesis disorders, such as the Zellweger syndrome, in which peroxisomes cannot be properly assembled [35]. Moreover, reduced plasmalogen levels have also been observed in several more common disorders like Alzheimer's disease [36–38], Parkinson's disease [39], Down syndrome [40], or schizophrenia [41] and have been suggested to constitute a part of the pathological mechanism during disease development.

Ethanolamine plasmalogens (PlsEtn, also called plasmenylethanolamines) and the diacyl glycerophospholipid PE (Fig. 1B) have an ethanolamine head group in common. To date, two main biosynthesis pathways for PE are known. The Kennedy pathway generates PE de novo and is crucial also for the final steps of PlsEtn production [42]. Here, PE synthesis starts from ethanolamine, which becomes phosphorylated and then activated by cytidine triphosphate (CTP). Cytidine diphosphate (CDP)-ethanolamine subsequently reacts with diacylglycerol completing the biosynthesis of PE [43]. Alternatively, PE can be produced via decarboxylation of PS at the inner mitochondrial membrane [44,45]. The importance of both of these pathways is stressed by the fact that targeted inactivation of either *Pcyt2*, the gene coding for the rate-limiting enzyme of the Kennedy pathway, or *Pisd*, the gene coding for PS decarboxylase, leads to embryonic death in mice [46,47]. Being the second most abundant phospholipid in eukaryotic cells, PE is essential for both structure and function of membranes. It is usually located at the cytoplasmic face of membranes, where, due to its cone shape, it supports the formation of non-lamellar structures. In addition, PE has been found to stabilize membrane proteins and to assist in their folding [48].

In the present study, we investigated how the level and side chain composition of PE respond to PlsEtn deficiency and to excess of PlsEtn induced by exogenous supplementation with precursors. To this end, we used fibroblasts derived from RCDP patients and gray matter brain tissue of *Gnpat* knockout mice as in vitro and in vivo models of ether lipid deficiency. The main objective was to determine how alterations in the PlsEtn concentration affect: (i) the total level of ethanolamine phospholipids (PE + PlsEtn); (ii) the total PUFA level; and (iii) the side chain composition at the *sn*-2 position of ethanolamine phospholipids.

## 2 Materials and methods

### 2.1 Patient phenotype classification

**Fig. 2:**
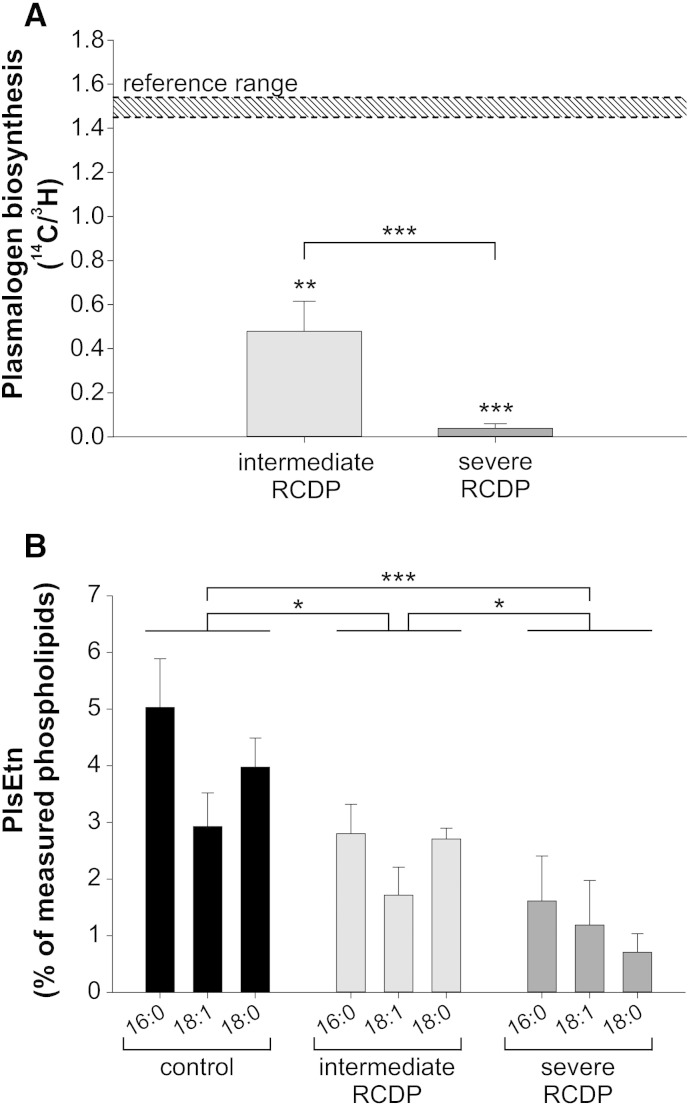
PlsEtn levels of cultured RCDP fibroblasts correlate with disease severity. (A) Plasmalogen biosynthesis rates were determined by the method of Roscher et al. [51] and are depicted as ratio between the peroxisomal (^14^C) and the ER (^3^H) steps of biosynthesis. Typical biosynthesis rates of healthy controls are indicated by the shaded area (“reference range”). ***P ≤ 0.001; **P ≤ 0.01 (one-sample *t*-test versus reference range; two-tailed *t*-test comparing intermediate and severe RCDP fibroblasts) (B) PlsEtn levels were measured in primary human fibroblasts. PlsEtn subspecies are grouped according to their different *sn*-1 moieties indicated by 16:0, 18:0 and 18:1. Data are represented as means ± SD derived from at least three different cell lines per group. ***P ≤ 0.001; *P ≤ 0.05 (one-way ANOVA with Tukey's post hoc test for comparison of total plasmalogen levels).

Clinical and biochemical phenotypes of patients, whose cells were studied here, have been described previously [32,49,50]. Phenotype severity classes were based on clinical features, red blood cell (RBC) plasmalogen levels and plasmalogen synthesis in patient fibroblasts (Fig. 2A and Table 1). Patients were classified as either severe or intermediate RCDP. Compared with severe RCDP, the intermediate form is characterized by improved growth and development and may not involve rhizomelia. In the intermediate form, residual plasmalogen synthesis and RBC plasmalogen levels are at least 30% of the control mean and more than two standard deviations above the severe RCDP mean.

### 2.2 Determination of plasmalogen biosynthesis rate and RBC plasmalogens

Plasmalogen biosynthesis rates and RBC plasmalogen levels were determined by established methods in the Peroxisomal Diseases Laboratory at the Kennedy Krieger Institute [51–53]. Briefly, for the measurement of plasmalogen biosynthesis, cultured cells were incubated with [^14^C]-hexadecanol and 1-O-[9, 10-^3^H]-hexadecylglycerol. Lipids were separated by thin layer chromatography and the ^14^C/^3^H ratio in plasmalogens determined. Incorporation of the ^14^C substrate requires the peroxisome while that of the tritiated substrate bypasses the peroxisomal steps and requires ER reactions, thereby serving as an internal control. Thus, the ratio of ^14^C to ^3^H incorporation into plasmalogens provides an accurate and reproducible measure of the peroxisomal ether lipid biosynthesis steps.

To determine RBC plasmalogens, lipids were extracted from packed RBCs derived from EDTA whole blood using isopropanol/hexane (2:3 vol/vol). The lipid layer was dried under a stream of nitrogen and exposed to 1 M methanolic HCl at 75 °C overnight to yield fatty acid methyl esters (FAME) from complex lipids and dimethylacetals (DMA) from plasmalogens. FAME and DMA were extracted from acidic methanol with hexane and analyzed by capillary gas chromatography. Both were referred to internal standards and results presented as ratios C16:0 DMA/C16:0 FAME.

### 2.3 Cell culture

Mutated genes (determining the RCDP subtype) and mutations of human primary RCDP skin fibroblasts are summarized in Table 1. Informed consent for the use of patient cell lines for research was obtained from the Johns Hopkins Hospital (Baltimore, USA) and the McGill University Health Center according to institutional guidelines. Control fibroblasts derived from metabolically healthy individuals were obtained from the General Hospital of Vienna (AKH). All studies involving human fibroblasts were approved by the Ethical Review Board of the Medical University of Vienna (application no. 729/2010).

Fibroblasts were maintained at 37 °C and 5% CO_2_ in RPMI-1640 medium (Lonza) supplied with 10% fetal bovine serum (FBS) (PAA), 2 mM l-glutamine (Lonza), 50 units/ml penicillin, 100 μg/ml streptomycin (Invitrogen) and 0.5% fungizone (Invitrogen). For application of plasmalogen precursors, fibroblasts were supplemented with 20 μM 1-O-hexadecylglycerol (Alexis Biochemicals) or batyl alcohol (1-O-octadecylglycerol, Sigma), each dissolved in ethanol, for three subsequent days with change of medium every 24 h.

### 2.4 Mice

Mice with a targeted inactivation (knockout) of the *Gnpat* gene (*Gnpat^tm1Just^*) have been described previously [19]. The strain was maintained on an outbred C57BL/6 × CD1 background and experimental cohorts with *Gnpat*^−/−^ (ko) and *Gnpat*^+/+^ (wt) littermates were obtained by mating heterozygous animals. Genotypes were determined at weaning by PCR as described previously [19] and confirmed after sacrifice. Mice were fed standard chow with food and water ad libitum and were housed in a temperature- and humidity-controlled room with 12:12 h light–dark cycle and a low level of acoustic background noise at the local animal facility of the Medical University of Vienna. All animals received humane care and handling in compliance with the national (Austrian) regulations (BGBl. II Nr. 522/2012) and the directive 2010/63/EU of the European Parliament and the council of the European Union.

### 2.5 Homogenization of cultured cells and murine brain tissue

For homogenization of cultured cells, around 10^6^ cells were washed with sterile PBS and distilled water (dH_2_O) and subsequently scraped into 1 ml of dH_2_O. Homogenates were obtained by passing through 23-G (2 passages), 25-G (2 passages) and 27-G (3 passages) needles.

For homogenization of brain material, mice were sacrificed by CO_2_ inhalation and their brains removed. To obtain gray matter samples, cerebral cortex and hippocampus were dissected and combined before homogenizing in dH_2_O (100 mg tissue/ml) supplied with cOmplete™ protease inhibitor cocktail (Roche Applied Science) using a glass–Teflon tissue grinder (Potter-Elvehjem homogenizer) at 4 °C. All homogenates were stored in aliquots at -80 °C until further use.

### 2.6 Determination of phospholipids

Lipid extractions were performed according to Bligh and Dyer [54]. Briefly, to 500 μl aqueous sample in a 10 ml Wheaton vial with Teflon-screw cap 3.75 vol of CHCl_3_/MeOH (5:10; vol/vol) were added and vortexed for 5 min, followed by the stepwise addition of 500 μl CHCl_3_ and 500 μl H_2_O, with 5 min vortex mixing after each step. Samples were centrifuged for 5 min at 500 × *g* to facilitate phase separation. The lower organic phase was transferred with a glass pipette to a new Wheaton vial containing 500 μl H_2_O, while the upper phase was re-extracted with 500 μl CHCl_3_. After vortex mixing and centrifugation, the lower phase of the vial, to which H_2_O was added, was transferred to a new Wheaton vial, and the lower phase from the CHCl_3_ re-extraction was transferred to the vial containing the residual H_2_O phase. Following vortex mixing and centrifugation, the lower phase was added to the lower phase from the first H_2_O wash and the combined CHCl_3_ phases were subjected to solvent evaporation under a gentle nitrogen flow at 37 °C. Lipid films were resuspended in mass spectrometry (MS) solvent or were directly used for phosphate determinations [55].

For mass spectrometry, 4 nmol phospholipids per sample were extracted in the presence of a lipid standard mix containing 33 pmol PlsEtn-Mix 1 (16:0p/15:0, 16:0p/19:0, 16:0p/25:0), 46.5 pmol PlsEtn-Mix 2 (18:0p/15:0, 18:0p/19:0, 18:0p/25:0), 64.5 pmol PlsEtn-Mix 3 (18:1p/15:0, 18:1p/19:0, 18:1p/25:0), 100 pmol PC (26:0, 28:0, 40:0 and 42:0), 100 pmol SM (d18:1/14:0, d18:1/17:0 and d18:1/25:0), 50 pmol PE (28:2, 40:2 and 44:2) and 50 pmol PS (28:2, 40:2 and 44:2). PE and PS [56], PlsEtn [57] and SM [58] standards were synthesized and purified via HPLC as described. PC standards were purchased from Avanti Polar Lipids. Dried lipids were dissolved in 100–200 μl methanol containing 10 mM ammonium acetate. Mass spectrometric analyses were done on a triple quadrupole instrument (Quattro II, Micromass) equipped with a nano-electrospray ionization (ESI) source. Nano flow tips were purchased from Teer Coatings. The source temperature was set to 30 °C and a capillary voltage of ± 600–900 V and a cone voltage of 30 V were applied, depending on the ion mode. Argon was used as collision gas at a nominal pressure of 2.5 × 10^− 3^ mbar. PC and SM species were analyzed by precursor ion scanning in positive ion mode, selecting for fragment ions of 184 Da (collision energy of 32 eV). PlsEtn quantitation was performed by precursor ion scanning in positive ion mode, selecting for fragment ions of 364 Da (16:0p species), 390 Da (18:1p species), and 392 Da (18:0p species), with a collision energy of 18–20 eV. PS and PE species were detected by neutral loss scanning, selecting for a neutral loss of 141 Da or 185 Da (positive ion mode), respectively, with a collision energy of 20 eV. Peak lists were corrected for C13 isotope effects and for mass-dependent changes in the response as described [59]. Lipid data are presented as mole% of measured phospholipids. Lipid species were annotated as follows: lipid category_ total number of C-atoms in the fatty acyl chains:number of double bonds.

### 2.7 Data analysis and statistical testing

A summary of all measured phospholipid species is provided in Supplementary Table 1. The following species were added to the group of PUFA-containing PE: 36:5, 36:4, 36:3, 38:6, 38:5, 38:4, 38:3, 40:7, 40:6, 40:5, 40:4, 40:3. PE 36:2 and 38:2 were not included, as, at least in mouse cerebellum, these species were found to almost exclusively consist of monounsaturated fatty acid chains [60]. Furthermore, both represent minor species and their inclusion would not cause any noticeable change in the amount of PUFA-containing PE. In order to estimate the amounts of arachidonic acid (AA) and docosahexaenoic acid (DHA) in PE, subspecies were grouped according to the number of double bonds in their side chains at the *sn-1* and *sn-2* position. In agreement with recent literature, we assumed that species with four or five double bonds usually contain AA, whereas species with six or seven double bonds usually involve a DHA side chain [60].

In general, all phospholipid determinations involving cultured cells were done in triplicates (three samples per cell line). The means of these triplicates were used to form the groups of control, intermediate RCDP and severe RCDP fibroblasts. All error bars in the graphs reflect standard deviations. Statistical analysis was performed using Sigma Plot 12.0 (Systat Software) and PASW Statistics 18.0 (SPSS Inc.). Plasmalogen biosynthesis rates of intermediate and severe RCDP fibroblasts were compared with each other by two-tailed *t*-test and with the reference range by one-sample *t*-tests. Results involving control, intermediate and severe RCDP fibroblasts were statistically evaluated by one-way analysis of variance (ANOVA) with Tukey's post hoc test. Where linear trends between the three groups were examined, unweighted linear terms were calculated in the course of one-way ANOVA. Also, planned contrasts (one-tailed) were used to compare controls with both RCDP groups in terms of fatty acid species in PE. In case of treatment experiments, statistical analysis was done by one-way ANOVA with Dunnett's post hoc test using ethanol-treated control cells as reference. Results involving wild type and *Gnpat* knockout mice were analyzed by two-tailed *t*-tests.

*P* values derived from post-hoc testing after ANOVA and from *t*-tests were adjusted for multiple testing in case of control lipid classes (PC, SM, PS) and fatty acid species at the *sn-2* position of plasmalogens using Bonferroni–Holm correction.

## 3 Results

### 3.1 Disease severity is reflected by the amount of PlsEtn in primary fibroblasts of RCDP patients

In order to assess changes in the lipid profile as a consequence of plasmalogen deficiency, we quantified different lipid species in primary skin fibroblasts derived from RCDP patients and, for comparison, from healthy controls using nano-mass spectrometry (Supplementary Table 1). Patient fibroblasts represented all three types of RCDP, including three cell lines of RCDP type 1 and two lines each of RCDP type 2 and type 3 (Table 1). First, we compared plasmalogen biosynthesis rates of the different fibroblasts. To this end, cultured cells were supplied with radiolabeled hexadecanol (^14^C) and hexadecylglycerol (^3^H), which are both metabolized to plasmalogens. Whereas hexadecanol depends on peroxisomes to be incorporated into plasmalogens, hexadecylglycerol bypasses the peroxisomal steps and plasmalogen biosynthesis fully proceeds at the ER. Consequently, the incorporation ratio of the two labels can be used as a measure of the peroxisomal contribution to plasmalogen biosynthesis activity. Independent of the type of RCDP, which is defined by the genotype, biosynthesis rates were in good agreement with the clinical disease severity of the respective patients. When the RCDP lines were grouped according to the course of the disease, the activity ratios differed strongly from each other. Cells from patients with intermediate RCDP showed clearly impaired plasmalogen biosynthesis compared with controls, whereas in cells from patients with severe RCDP hardly any biosynthesis activity could be detected (Fig. 2A). Accordingly, for further analyses, the RCDP fibroblast lines were classified into intermediate (n = 3) and severe (n = 4) cases.

Next, we performed a quantification of PlsEtn levels using nano-electrospray ionization tandem mass spectrometry (nano-ESI–MS/MS), which revealed that the severity of disease in RCDP patients was also reflected by the residual amount of PlsEtn in the cultured fibroblasts. Compared with control lines, fibroblasts of the intermediately affected group were found to have about 40% reduced levels of PlsEtn, while those derived from patients with severe phenotype displayed a more pronounced reduction of more than 70% representing a highly significant linear trend between the three examined groups (*P* < 0.001). This decrease affected all plasmalogen subtypes, irrespective of their *sn*-1 chain (Fig. 2B). C16:0 turned out to be the most abundant species in controls as well as in both RCDP groups. Compared with the intermediate RCDP group, the residual PlsEtn in fibroblasts of severely affected patients was more strongly depleted in C18:0 than in the two other species (Fig. 2B). Furthermore, PlsEtn levels of cultured fibroblasts clearly correlated with those in red blood cells of the corresponding patients (Table 1). Although plasmalogen levels in patient fibroblasts were in accordance with their rate of biosynthesis, there was an unexpected residual amount of PlsEtn in fibroblasts of the severely affected group. Residual plasmalogens in different types of cultured ether phospholipid-deficient cells were also reported by others [61–63]. Furthermore, we made similar observations in embryonic fibroblasts derived from *Gnpat* knockout mice (data not shown), in which plasmalogens are virtually absent in vivo [19]. Therefore, residual plasmalogens – or their precursors – most likely are derived from the serum added to the culture medium. Attempts to avoid this uptake by the use of synthetic medium without serum were unsuccessful due to reduced viability and a general change in the phospholipid profile (data not shown). Thus, we retained our original culture conditions to allow for comparison of our results with previous studies.

### 3.2 PE strictly compensates for plasmalogen deficiency

**Fig. 3:**
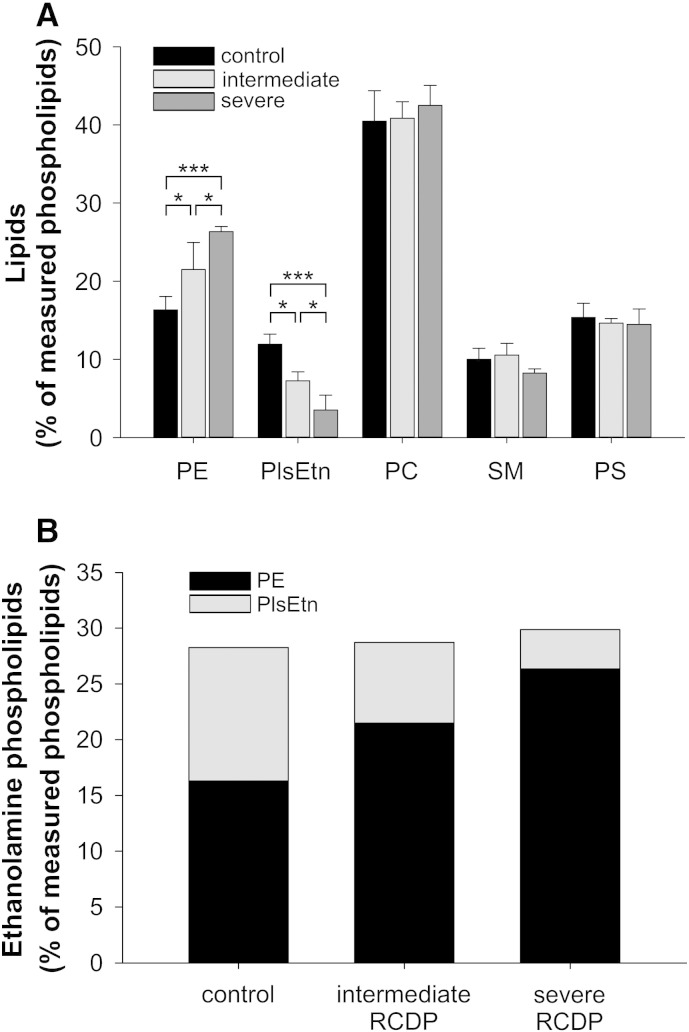
Total ethanolamine phospholipid levels are precisely maintained upon PlsEtn deficiency. (A) The levels of the main phospholipid classes were determined in primary human fibroblasts derived from healthy controls, intermediately and severely affected RCDP patients by mass spectrometry. Data represent means ± SD of at least three different cell lines per group. ***P ≤ 0.001; *P ≤ 0.05 (one-way ANOVA with Tukey's post hoc test; Bonferroni–Holm correction for multiple testing in control lipid classes) (B) Total ethanolamine phospholipid levels are depicted as stacked bars composed of PlsEtn and PE levels. Data consist of mean values for the two lipids as shown in A. *P* ≥ 0.60 for all comparisons of total ethanolamine phospholipid levels (one-way ANOVA with Tukey's post hoc test). PE, phosphatidylethanolamine; PlsEtn, ethanolamine plasmalogen; PC, phosphatidylcholine; SM, sphingomyelin; PS, phosphatidylserine.

In order to investigate compensatory alterations of the lipid profile in response to decreased plasmalogen levels, we analyzed the main phospholipid classes in control and RCDP fibroblasts. In particular, we were interested in how different degrees of plasmalogen reduction affect potential compensatory mechanisms. Consistent with previous reports in other cell types or tissues [19,64–67], the reduction of PlsEtn in RCDP fibroblasts was accompanied by a rise in PE. All other major classes of phospholipids, including PC, SM and PS, remained at the level of controls (Fig. 3A). PE levels adapted conversely to the degree of plasmalogen deficiency, keeping the total amount of ethanolamine-containing phospholipids remarkably constant, slightly below 30% of total phospholipids (Fig. 3B). While in controls almost half of all ethanolamine phospholipids was PlsEtn, PE accounted for almost 75% and 90% of ethanolamine species in the fibroblasts of intermediately and severely affected patients, respectively. The continuous increase of PE over the three groups was also reflected statistically by a highly significant linear trend (*P* < 0.001).

### 3.3 Compensation for plasmalogen deficiency is mainly accomplished through arachidonic acid-containing, but not docosahexaenoic acid-containing PE species in human fibroblasts

**Fig. 4:**
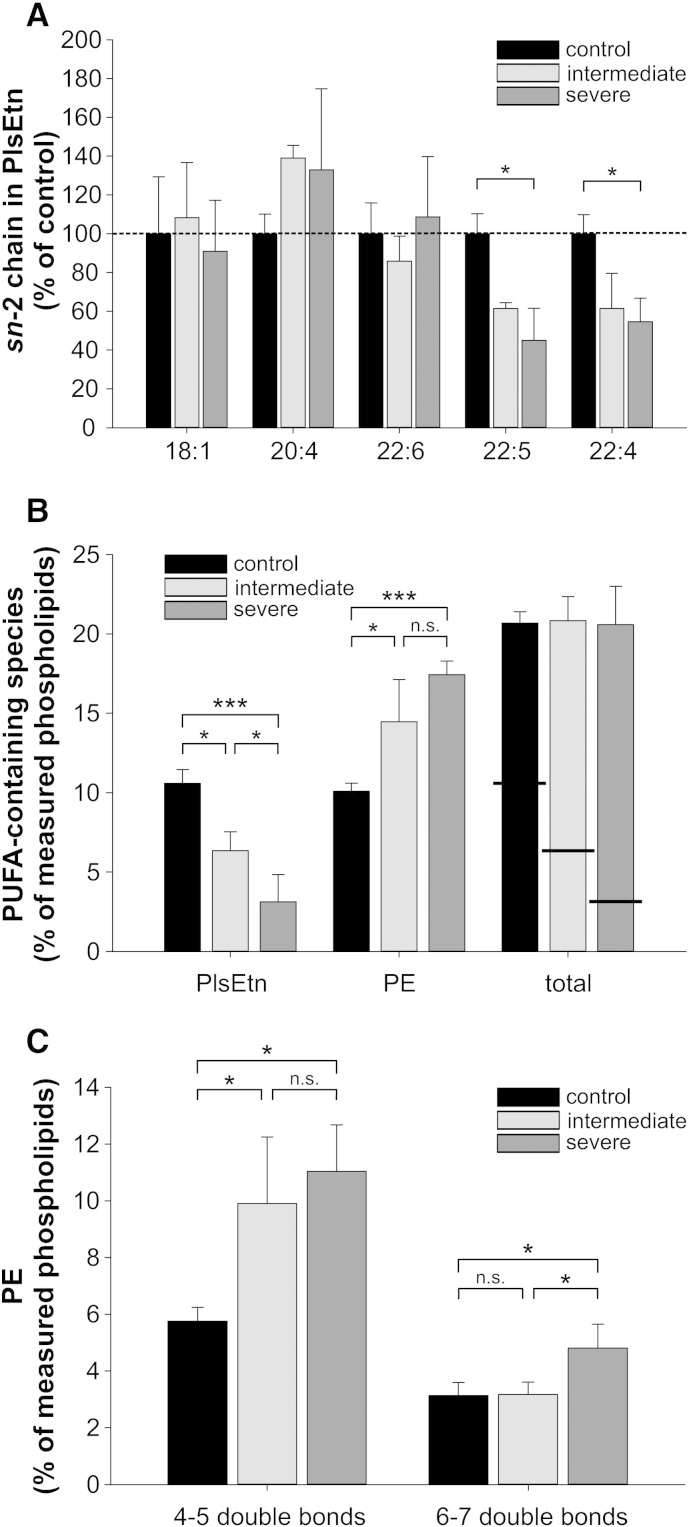
Ethanolamine phospholipids of RCDP fibroblasts have normal levels of total PUFAs but are enriched in arachidonic acid. (A) The composition of the *sn*-2 side chains was determined in PlsEtn of the different fibroblast groups and the concentrations of the individual species were calculated as percentage of total PlsEtn. To enable better comparison, relative values for the most abundant species are shown with those of the control group normalized to 100%. Values not normalized to controls for all measured species are shown in Supp. Fig. 1. *P ≤ 0.05 (one-way ANOVA with Tukey's post hoc test; Bonferroni–Holm correction for multiple testing) (B) PUFA side chains were added up in PlsEtn and PE. Horizontal lines in the bars representing total PUFA species indicate the different contributions of PE and PlsEtn. Individual species contributing to the PUFA group are listed in Section 2.7. ***P ≤ 0.001; *P ≤ 0.05; *n.s.*, not significant (one-way ANOVA with Tukey's post hoc test). *P* ≥ 0.98 for all comparisons of total PUFA levels (C) PE species were grouped according to the number of double bonds in their side chains into PE with 4–5 double bonds (mostly AA-containing) or PE with 6–7 double bonds (mostly DHA-containing). Data represent means ± SD of at least three different cell lines. ***P ≤ 0.001; *P ≤ 0.05; *n.s.*, not significant (one-way ANOVA with Tukey's post hoc test).

Both PlsEtn and PE are rich in PUFAs; among these, arachidonic acid (AA, C20:4ω-6) and docosahexaenoic acid (DHA, C22:6ω-3) are the most abundant. In order to investigate if the total amount of PUFAs and also the ratio between AA and DHA are maintained upon compensation of plasmalogen deficiency by PE, we analyzed the fatty acid composition of PlsEtn and PE in control and RCDP fibroblasts. At the *sn*-2 position of PlsEtn, the major difference between the fibroblast groups was an increased frequency of AA (though not statistically significant) replacing C22:5 and C22:4 in both RCDP fibroblast groups compared with controls (Fig. 4A and Supp. Fig. 1). Interestingly, both latter species changed in a similar way and magnitude, and a clear linear trend from controls to severe RCDP fibroblasts was observed (*P* = 0.001 for C22:5 and *P* = 0.003 for C22:4). Whereas C22:4 is normally exclusively of the ω-6 type, C22:5 can be either ω-3 (serving as a precursor for DHA) or ω-6 (derived from the metabolization of C22:4) [68,69]. As C22:4 (ω-6) levels were found to be lowered in RCDP fibroblasts, whereas DHA (ω-3) remained unchanged, it is tempting to speculate that the reduced occurrence of C22:5 is mainly attributable to a decrease in the ω-6 component. The abundance of AA at the expense of C22:5 and C22:4 seems unlikely to be due to plasmalogens or their precursors present in the culture medium, as it was not restricted to the severely affected fibroblasts, but also found in cells of patients with intermediate disease course, where residual plasmalogen biosynthesis contributes to the PlsEtn content.

When comparing the total amount of PUFAs in the two ethanolamine phospholipids (PE and PlsEtn), we found that compensation for plasmalogen deficiency was also reflected in the levels of these essential fatty acids. The total amount of PUFAs incorporated into ethanolamine phospholipids was equal in controls and fibroblasts of patients with intermediate and severe RCDP (Fig. 4B), indicating a strict adaptation of the fibroblasts to the condition of ether phospholipid deficiency also in terms of PUFA side chains. This finding is underlined by the observed linear trends for the amounts of PUFAs in both phospholipid species: a decreasing one in PlsEtn (*P* < 0.001) and an increasing one in PE (*P* < 0.001).

Subsequently, we investigated whether PE species containing the two main PUFAs, AA and DHA, increase to a similar extent in RCDP fibroblasts. Because our mode of analysis of PE produced the sum of both fatty acid chains (e.g. 38:4), we grouped the PE species into those containing 4–5 and 6–7 double bonds. According to recent lipidomic studies, the former predominantly contain AA and the latter DHA [60]. Our analysis revealed that PlsEtn deficiency was almost exclusively compensated for by PE containing fatty acids with a total of 4–5 double bonds (AA-containing). Compared with controls, these species were almost doubled in concentration in both of the RCDP fibroblast groups. In contrast, the proportion of PE containing fatty acids with 6–7 double bonds (DHA-containing) was not altered in intermediately affected RCDP fibroblasts and only slightly increased in severely affected cells (Fig. 4C). A comparison of controls with both RCDP groups revealed a highly significant difference in the species with 4–5 double bonds (*P* = 0.005 using planned contrasts), but not in species with 6–7 double bonds (*P* = 0.097). In total, these results indicate that compensatory responses to plasmalogen deficiency predominantly involve AA, but not DHA, thus leading to a remarkable shift in the ratio between these two essential PUFAs.

### 3.4 PE levels in plasmalogen-deficient fibroblasts dynamically adapt to exogenous precursor supplementation

**Fig. 5:**
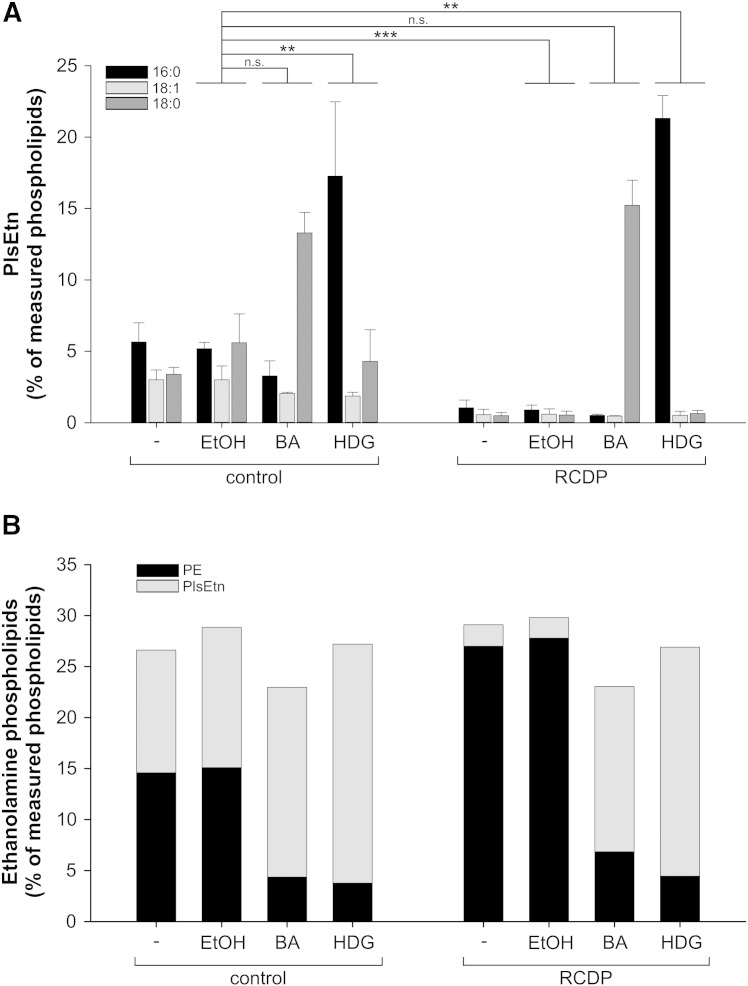
PE levels drop in response to excessive plasmalogen levels. (A) A control fibroblast line and a line derived from an RCDP patient with severe disease course were left untreated or were treated with solvent (ethanol, EtOH) or with plasmalogen precursor substances (BA, batyl alcohol; HDG, hexadecylglycerol). PlsEtn levels were determined and displayed according to their different *sn*-1 moieties (16:0, 18:1 and 18:0). Data are shown as means ± SD of three independent experiments. ***P ≤ 0.001; **P ≤ 0.01; *n.s.*, not significant (one-way ANOVA with Dunnett's post hoc test) (B) Total ethanolamine phospholipid levels of untreated, solvent-treated and plasmalogen precursor-treated fibroblasts are depicted as stacked bars composed of PlsEtn and PE levels. Data consist of mean values for the two lipids as shown in A. *P* ≥ 0.16 for all comparisons of total ethanolamine phospholipid levels (one-way ANOVA with Dunnett's post hoc test).

Deficiency in plasmalogen biosynthesis can be overcome in vitro and in vivo, with the exception of the brain, by dietary application of precursor substances that circumvent the peroxisomal biosynthesis steps [70,71]. In order to investigate their lipid composition under conditions of excess of plasmalogens, we treated RCDP (RCDP 1–3, cf. Table 1) and control fibroblasts with the ether phospholipid precursors batyl alcohol (BA; octadecylglycerol) or hexadecylglycerol (HDG), which are O-alkylglycerols with an ether-bonded C18:0 and C16:0 chain, respectively, at the *sn*-1 position. After three days of plasmalogen precursor treatment, the levels of PlsEtn were restored also in severely affected RCDP cells and even significantly exceeded the control values reaching 1.5 times (BA) or even twice (HDG) the amounts of untreated control cells. A similar rise in PlsEtn was also observed in treated controls (Fig. 5A). As expected and in line with previous studies involving plasmalogen precursor treatment [7,72,73], we did not find any side chain remodeling at the *sn*-1 position. As a consequence, only one of the three main PlsEtn species was rescued by the supplementation with BA or HDG, namely the C18:0 and C16:0 species, respectively. The two other species (C16:0, C18:1 and C18:0, C18:1 in case of BA and HDG treatment, respectively) remained at very low levels in the treated RCDP fibroblasts (Fig. 5A).

We also examined the overall phospholipid profile of the fibroblasts upon PlsEtn restoration by precursor supplementation. Consistent with the above findings in fibroblasts of RCDP patients with severe and intermediate disease course (Fig. 3), both BA and HDG supplementation had a strong effect on PE levels, whereas the levels of the other main phospholipid classes remained grossly unchanged, except for a small reduction in the levels of PC seen after HDG treatment in controls (*P* < 0.001) and also, by trend, in RCDP fibroblasts (*P* = 0.052 after adjustment for multiple testing; Supp. Fig. 2). Similar to under conditions of plasmalogen deficiency, alkylglycerol treatment led to adaptation of PE levels inversely proportional to the amount of PlsEtn. Total ethanolamine phospholipid homeostasis was maintained with PlsEtn constituting the major portion and PE accounting for only 17–30% of total ethanolamine species (Fig. 5B).

**Fig. 6:**
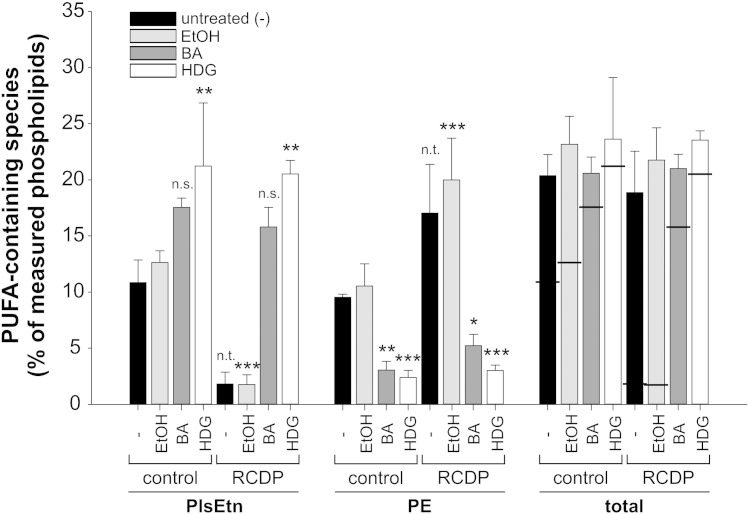
The total amount of PUFAs in ethanolamine phospholipids is maintained regardless of plasmalogen precursor status. The total amounts of PUFA side chains in PlsEtn and PE of untreated, solvent-treated or plasmalogen precursor-treated fibroblasts were determined. Horizontal lines in the bars representing total PUFA species indicate the different contributions of PE and PlsEtn. Individual species contributing to the PUFA group are listed in Section 2.7. Data represent means ± SD of at least three different cell lines. ***P ≤ 0.001; **P ≤ 0.01; *P ≤ 0.05; *n.s.*, not significant; *n.t.*, not tested (one-way ANOVA with Dunnett's post hoc test). *P* ≥ 0.71 for all comparisons of total PUFA levels. BA, batyl alcohol; HDG, hexadecylglycerol.

Both BA and HDG have been suggested for the treatment of human ether phospholipid deficiency [21,71,74,75]. When evaluating their potency, it is crucial to consider the restoration of both plasmalogen levels and the broad range of *sn*-2-positioned PUFAs. Therefore, we also determined the total amount of PUFAs in both ethanolamine phospholipid species. In controls as well as in RCDP fibroblasts, precursor-treated and untreated, the level of PUFAs was kept constant (Fig. 6) indicating that, independent of the type of ethanolamine phospholipid, PUFAs are incorporated in strictly maintained amounts. Moreover, the distribution of individual fatty acid species at the *sn*-2 position after treatment was nearly identical to that in control fibroblasts (data not shown).

### 3.5 PE of murine gray matter tissue similarly adapts to plasmalogen deficiency

**Fig. 7:**
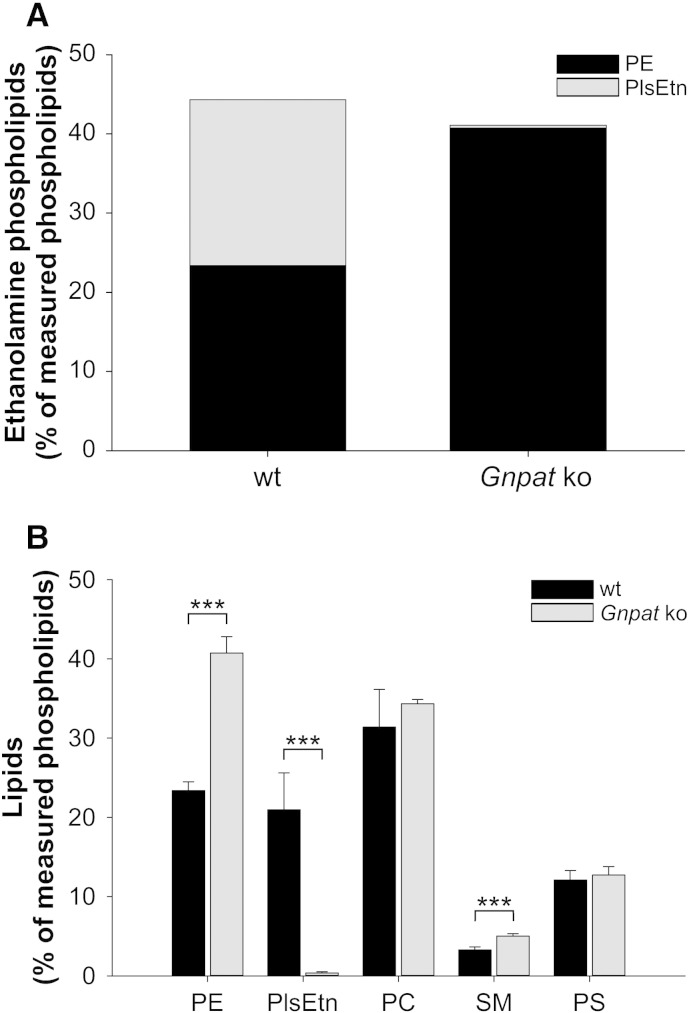
Total ethanolamine phospholipid levels are maintained also in the plasmalogen-deficient murine brain. Gray matter tissue (cerebral cortex and hippocampus) was dissected from wild type (wt, n = 10) and *Gnpat* knockout (ko, n = 4) mice for quantitative analysis of the main phospholipid classes. (A) Total ethanolamine phospholipid levels are depicted as stacked bars composed of mean values of the PlsEtn and PE levels. *P* = 0.227 (two-tailed *t*-test) (B) The levels of all major phospholipid classes are shown as means ± SD of all mice examined. ***P ≤ 0.001 (two-tailed *t*-test; Bonferroni–Holm correction for multiple testing in control lipid classes). PE, phosphatidylethanolamine; PlsEtn, ethanolamine plasmalogen; PC, phosphatidylcholine; SM, sphingomyelin; PS, phosphatidylserine.

The *Gnpat* knockout mouse, in which the first enzyme in the ether lipid biosynthesis pathway is inactivated, serves as a model of RCDP [19]. As a consequence of their inability to synthesize ether lipids, these animals show symptoms reminiscent of RCDP like growth and mental retardation, defective myelination, visual impairments, infertility and a reduced lifespan [19,76,77]. In human gray matter of the brain, more than 50% of ethanolamine phospholipids are present as plasmalogens [37]; therefore, we analyzed the extent of compensation for PlsEtn deficiency in vivo in cerebral gray matter of *Gnpat* knockout mice. As expected, PlsEtn was hardly detected in the tissue of *Gnpat* knockout mice, whereas in wild type gray matter it accounted for almost 50% of ethanolamine phospholipids, corresponding to about 20% of total phospholipids. C18:0 was the most prevalent *sn*-1 species, followed by C18:1 and C16:0 (Supp. Fig. 3). Complete PlsEtn deficiency was accompanied by a pronounced increase in PE levels, such that PE in *Gnpat* knockout mice amounted to the sum of ethanolamine phospholipids (PlsEtn + PE) in wild type controls (Fig. 7A). These results strongly confirm our in vitro findings indicating that similar homeostatic mechanisms maintain total ethanolamine phospholipid levels also in vivo. As in RCDP fibroblasts, in the murine brain PE was by far the phospholipid contributing the most to the compensatory adaptation, while other main lipid classes basically remained unchanged except for a minor, but statistically significant elevation of SM levels in *Gnpat* knockout mice (Fig. 7B).

**Fig. 8:**
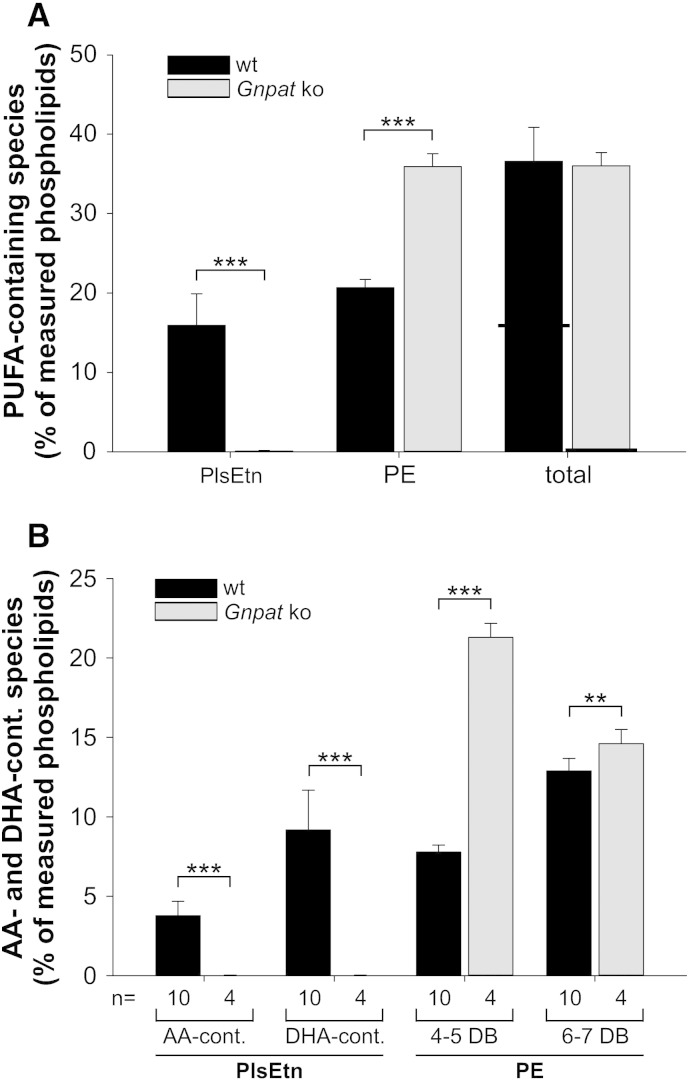
PUFA side chain analysis in mouse tissue confirms that AA is enriched at the expense of DHA in plasmalogen deficiency also in vivo. (A) PUFA side chains were added up in PlsEtn and PE derived from wild type and *Gnpat* knockout (ko) gray matter tissue. Horizontal lines in the bars representing total PUFA species indicate the different contributions of PE and PlsEtn. Individual species contributing to the PUFA group are listed in Section 2.7. ***P ≤ 0.001 (two-tailed *t*-test) (B) PlsEtn species were grouped into AA-containing and DHA-containing types, whereas PE species were grouped into such with 4–5 double bonds (mostly AA-containing) or such with 6–7 double bonds (mostly DHA-containing). Data represent means ± SD of 10 (wt) and 4 (*Gnpat* ko) animals per group. ***P ≤ 0.001; **P ≤ 0.01 (two-tailed *t*-test). DB, double bonds.

To evaluate the compensatory mechanism in greater detail, we again focused on the *sn*-2 position of both ethanolamine phospholipids. Like in human fibroblasts, also in murine gray matter, PUFAs in PE compensated for the lack of PUFAs in PlsEtn, leading to almost identical total PUFA amounts in ethanolamine phospholipids in ether lipid-deficient and wild type brain tissue (Fig. 8A). Although in wild type PlsEtn (and also PE) DHA was much more common than AA, compensatory upregulation was mainly restricted to predominantly AA-containing PE with 4–5 double bonds (Fig. 8B). These species increased from 8% of all measured phospholipids in wild type brains to 21% in the brains of knockout animals, whereas there was only a minimal increase in mostly DHA-containing PE with 6–7 double bonds. Remarkably, although this causes a massive change in the ratio between DHA and AA, there was no counteracting enrichment of DHA in any other main phospholipid class (data not shown).

Together with the results obtained in human fibroblasts, these data show that under conditions of plasmalogen deficiency, AA-containing ethanolamine phospholipids are strongly elevated at the expense of DHA-containing ethanolamine species. Thus, plasmalogen deficiency results in an altered ratio of ω-6 and ω-3 fatty acids.

## 4 Discussion

Plasmalogens are major essential constituents of all cellular membranes in mammals. Therefore, it appears evident that cells employ compensatory mechanisms to maintain an optimal phospholipid composition in order to cope with plasmalogen deficiency. In the present study, we found that the main compensatory response to the lack of PlsEtn is an upregulation of PE. Previous studies have reported a rise in PE levels as a consequence of plasmalogen deficiency [19,64–67]. Here, we extend this observation and show that the amounts of total ethanolamine phospholipids are accurately regulated and sustained at a remarkably constant level under conditions of complete or partial absence as well as upon excess of plasmalogens in vitro and in vivo. Dynamic adaptation of cellular phospholipid composition to exogenous lipid alterations has previously been shown in other model systems, for example, PC and PE adapt to changes in membrane cholesterol levels [78,79]. Here, we show that PE, but not the other major membrane phospholipids, underlies a tight inverse adjustment in response to the altered levels of PlsEtn.

Based on our results, the question arises, how such an accurate regulation of total ethanolamine phospholipids is accomplished. Two major different pathways contribute to PE biosynthesis, which both could potentially be involved in the homeostatic regulation of PE. The first, the Kennedy pathway, appears as an obvious candidate, as the two ethanolamine phospholipid species share some steps of this biosynthesis pathway [42]. The production of both PE and PlsEtn depends on ethanolaminephosphotransferase (EC 2.7.8.1) for the coupling of CDP-ethanolamine to either diacylglycerol or alkylacylglycerol in the last step of the pathway. Under most physiological conditions, the generation of either CDP-ethanolamine or the lipid precursor is rate-limiting [80]. If the compensatory increase (upon PlsEtn deficiency) and decrease (upon ether lipid precursor supplementation) of PE derives from the Kennedy pathway, we would assume that adaptation results from the shift in the relative abundance of the two lipid precursors, diacylglycerol and alkylacylglycerol, while CDP-ethanolamine levels remain constant. Consequently, PE would be produced instead of PlsEtn in the case of alkylacylglycerol depletion and, conversely, under excess of alkylacylglycerol (as seen after plasmalogen precursor treatment), PlsEtn levels would increase at the expense of PE. Thus, our data are in good agreement with a regulation of total ethanolamine phospholipids by adjusting the concentration of CDP-ethanolamine generated by CTP:phosphoethanolamine cytidyltransferase (ECT, EC 2.7.7.14). In humans, this enzyme is encoded by the *PCYT2* gene. Knowledge about if and how ECT is regulated by phospholipid levels is limited, but recent studies in various cell lines showed transcriptional upregulation of the murine *Pcyt2* gene in response to serum depletion, that is a deprivation of lipids [81,82]. For the second possibility, it has been reported that, in cultured cells, PE biosynthesis is mainly dependent on PS decarboxylation [43,80]. This pathway, however, supposedly does not contribute to PlsEtn biosynthesis [42]. PE produced by PS decarboxylation differs from PE originating from the Kennedy pathway in that it contains a larger proportion of PUFAs [83]. Our findings that PUFA-rich PE compensates for plasmalogen deficiency, thus, are consistent with an involvement of PS decarboxylase. Unfortunately, very little is known about the regulation of PS decarboxylase activity. The possible existence of a feedback loop, which is controlled by total ethanolamine phospholipid levels, remains to be investigated. Currently, we can also not exclude that reduced degradation or turnover of PE contributes to the compensatory elevation.

A major function attributed to plasmalogens, especially in the central nervous system, is the storage of PUFAs [11,84]. In the present study we show that, even upon plasmalogen deficiency, the total amount of PUFAs in ethanolamine phospholipids is kept constant in human fibroblasts and in the murine brain. This is particularly intriguing since gray matter plasmalogens are specifically enriched in PUFAs. The maintenance of total PUFA levels is achieved by elevated levels of PE rich in PUFAs. A similar observation has been made in post mortem brain tissue of a case with Zellweger syndrome, where PUFA-containing PE proved to be increased [66]. However, in our models, the two major PUFA species, DHA and AA, appear to be unequally represented in the altered ethanolamine phospholipid fraction, pointing toward a selective enrichment of AA over DHA in response to plasmalogen deficiency. This observation is underlined by two findings: (i) residual PlsEtn in RCDP fibroblasts showed a trend toward increased proportions of AA at its *sn*-2 position; and (ii) in comparison to control fibroblasts or wild type brains, AA-containing PE species were strongly elevated in RCDP fibroblasts as well as in gray matter from *Gnpat* knockout brains, while DHA-containing PE largely remained the same. As a consequence, our results offer a plausible explanation for the previously observed general reduction of DHA in whole brain homogenates of *Gnpat* knockout mice [19]. Also in the brains of patients with peroxisome biogenesis disorders (Zellweger spectrum), a decrease in DHA in ethanolamine phospholipids was determined [85]. However, when comparing tissue or fibroblast levels of DHA of RCDP and Zellweger spectrum cases, it has to be taken into account that in the latter all peroxisomal functions are affected, including the generation of DHA from its precursor [86]. In addition, the increased amount of AA that we describe here, is in good agreement with a recent study in a plasmalogen-deficient murine macrophage cell line demonstrating the enhanced release of PE-bound AA upon stimulation [87].

All these observations raise the question as to the origin of the observed changes. We suggest two alternative possibilities; the enrichment of AA at the expense of DHA can be caused by either: (i) a difference in the availability of the two fatty acids; or (ii) a fortuitously or deliberately altered regulation under conditions of plasmalogen deficiency. Concerning the first alternative, it is widely accepted that the exogenous supply and the availability of fatty acids are reflected by the fatty acid composition of phospholipids [88,89]. However, in contrast to Zellweger spectrum disorders, it is not obvious, why fatty acid availability would be altered in case of isolated ether lipid deficiency. In the cultured cells, exogenous fatty acids are exclusively derived from the medium and AA might be enriched compared with DHA, as it is also the main fatty acid species in PE under healthy conditions (Fig. 4C). This does not hold true for the murine gray matter, where DHA dominates in wild type mice. In this case, PUFAs incorporated into PE could be derived from the circulation [89] and, therefore, the fatty acid composition of PE in the brain may reflect their availability in the periphery. This view is supported by the fact that in blood cells, PE is richer in AA than in DHA [90]. In the second possibility, the preferred integration of AA could result from an altered regulation caused by ether lipid deficiency, for example, in the production of diacylglycerol. It is also possible that AA serves a specific function in the absence of plasmalogens, such as providing distinct membrane properties or mediating signaling tasks. Irrespective of the underlying mechanism, plasmalogen deficiency appears to affect cellular functions by shifting the ratio between ω-6 (AA) and ω-3 (DHA) PUFAs.

In addition to the implications for the various inherited diseases associated with complete or partial plasmalogen deficiency, such as RCDP or Zellweger spectrum disorders, our findings may be of relevance also for more common disorders with less severe plasmalogen deficits, like Alzheimer's disease [36–38]. On the one hand, we show that deficiency of PlsEtn is rigorously counterbalanced by the rise in PE, implying that symptoms associated with these diseases are not the result of a general shortage of ethanolamine phospholipids. On the other hand, our results indicate that impaired biosynthesis of ether phospholipids precipitates a dual deficit, which is not compensated for by PE. One problem is the absence of phospholipids harboring the ether bond; the other is the decrease in DHA and the resulting dysbalance of ω-6 and ω-3 PUFAs. It is tempting to speculate about which phenotypic consequences derive from which deficit. The characteristic vinyl ether linkage attributes extraordinary biophysical properties to plasmalogens, for example by conferring membrane compaction and favoring membrane fusion [15,16,91]. Accordingly, impairments of membrane structure result from the deficiency of the ether moiety, even if ether lipids are replaced by their diacyl counterparts. Membrane compaction, for example, is a prerequisite in brain white matter myelin sheaths, which are affected by de- and dysmyelination in RCDP [92] or ether lipid-deficient mouse models [77]. Potentially, the vinyl ether bond has been suggested to exert antioxidative functions [15,67,93], which might be impaired by a reduced amount of plasmalogens, thus, inducing or aggravating oxidative stress. DHA, in turn, is known to be crucial for a variety of metabolic and immunologic processes [94]. In general, ω-6 and ω-3 fatty acids are metabolized to a large number of different immune mediators that serve pro- as well as anti-inflammatory functions [95]. As these two types of fatty acids cannot be mutually converted into each other, homeostasis of immunologic reactivity requires a constant ratio of ω-6/ω-3 PUFAs. As highlighted in a variety of nutritional studies, the permanent shift of this ratio toward ω-6 fatty acids is unfavorable and has been shown to be associated with cardiovascular disease or the development of inflammatory and neurodegenerative disorders, including Alzheimer's disease [96–98].

In conclusion, the present findings reveal extensive compensatory adaptations in the phospholipid composition, mainly in PE, in response to disturbances in PlsEtn metabolism. However, these homeostatic processes lead to a shift in the ratio of ω-6/ω-3 PUFAs. Thus, our study emphasizes the complexity of lipid homeostasis and the necessity to carefully consider these intricate compensatory mechanisms when dealing with possible consequences of plasmalogen depletion, or altered phospholipid composition in general, for different diseases and treatment strategies.

## Figures and Tables

**Table 1:** Summary of primary human skin fibroblasts derived from RCDP patients.

ID	Disease severity	RCDP type (mutated gene)	Mutation	RBC plasmalogens in patient (C16:0)^a^	Reference
RCDP 1-1	Intermediate	1 (*PEX7*)	H285R/L292X	0.031	[32]
RCDP 1-2	Severe	1 (*PEX7*)	H39P/W206X	0.002	[32]
RCDP 1-3	Severe	1 (*PEX7*)	L292X/L292X	0.001	[32,49]
RCDP 2-1	Intermediate	2 (*GNPAT*)	c. 1937 + 5G>A/c. 1937 + 5G>A	0.060	[49]
RCDP 2-2	Severe	2 (*GNPAT*)	c. 1428delC/c. 1428delC	0.005	[49,50]
RCDP 3-1	Intermediate	3 (*AGPS*)	E471K/E471K	0.028	[49]
RCDP 3-2	Severe	3 (*AGPS*)	T568M/T568M	0.003	[49]

a Reference values in controls: 0.065 ± 0.01; ratio of C16:0 dimethylacetals (representing plasmalogens) to C16:0 fatty acid methyl esters (representing other lipids).

## Appendix A Supplementary data

Supplementary material **Supplementary Figure 1.** The *sn*-2 position of PlsEtn in cultured human primary skin fibroblasts is mostly occupied by PUFAs. **Supplementary Figure 2**. The levels of non-ethanolamine phospholipids are hardly affected by plasmalogen precursor supplementation. **Supplementary Figure 3**. C18:0 is the major *sn*-1 species in PlsEtn in the murine wild type brain gray matter. **Supplementary Table 1**. Summary of detected phospholipid species.
