# Supplementary material for: Homeostasis of phospholipids — The level of phosphatidylethanolamine tightly adapts to changes in ethanolamine plasmalogens
Source: Biochim Biophys Acta. 2015 Feb;1851(2):117–28. doi: 10.1016/j.bbalip.2014.11.005 (PMC4331674; doi:10.1016/j.bbalip.2014.11.005)
Supplement: Supplementary material — Supplementary Figure 1. The sn-2 position of PlsEtn in cultured human primary skin fibroblasts is mostly occupied by PUFAs. Supplementary Figure 2. The levels of non-ethanolamine phospholipids are hardly affected by plasmalogen precursor supplementation. Supplementary Figure 3. C18:0 is the major sn-1 species in PlsEtn in the murine wild type brain gray matter. Supplementary Table 1. Summary of detected phospholipid species. [file mmc1.pdf]

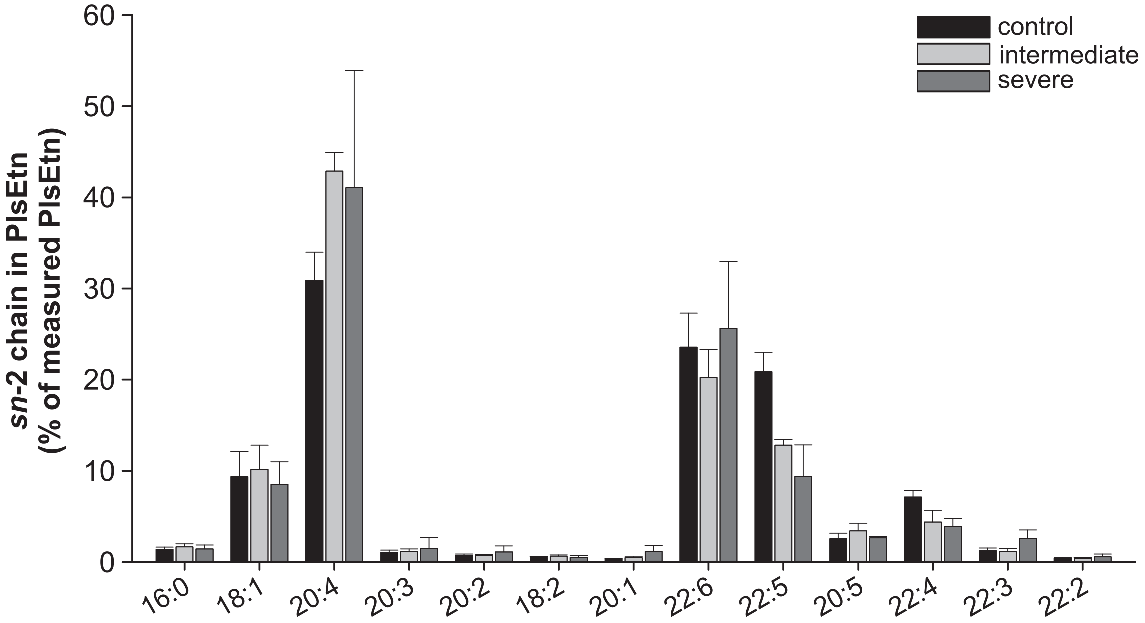

Supplementary Figure 1. ***The sn-2 position of PlsEtn in cultured human primary skin fibroblasts is mostly occupied by PUFAs.*** *Sn-2* side chains of PlsEtn in primary human fibroblasts are depicted as % of total measured PlsEtn. Mind the normalization to total PlsEtn due to the low levels in RCDP cells. For values normalized to controls, refer to Fig. 4A. Data represent means ± SD of at least three different cell lines.

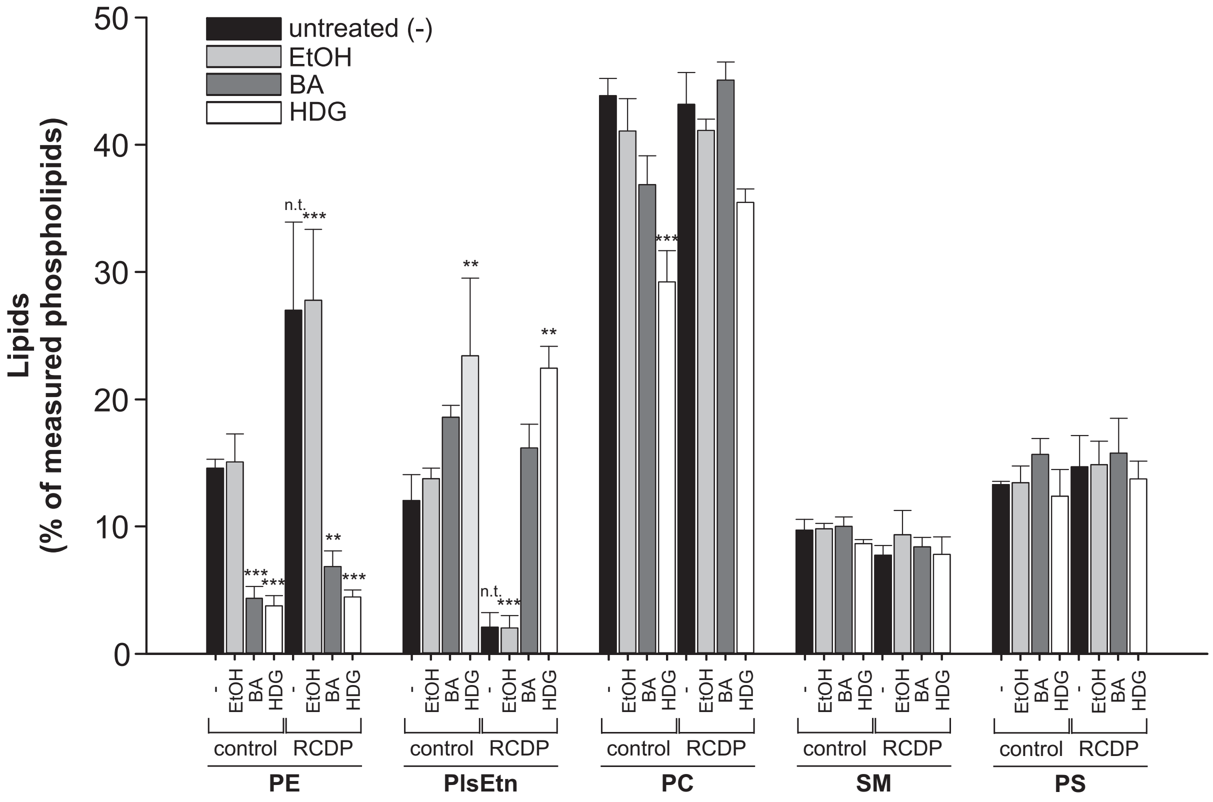

Supplementary Figure 2. ***The levels of non-ethanolamine phospholipids are hardly affected by plasmalogen precursor supplementation.*** The levels of the main phospholipid classes were determined in untreated, solvent-treated or plasmalogen precursor-treated primary human fibroblasts derived from a control and a severely affected RCDP patient. Data shown are means ± SD of three independent experiments. ****P* ⩽ 0.001; ***P* ⩽ 0.01 (one-way ANOVA with Dunnett's post hoc test; Bonferroni-Holm correction for multiple testing in control lipid classes). BA, batyl alcohol; HDG, hexadecylglycerol; n.t., not tested; PE, phosphatidylethanolamine; PlsEtn, ethanolamine plasmalogen; PC, phosphatidylcholine; SM, sphingomyelin; PS, phosphatidylserine

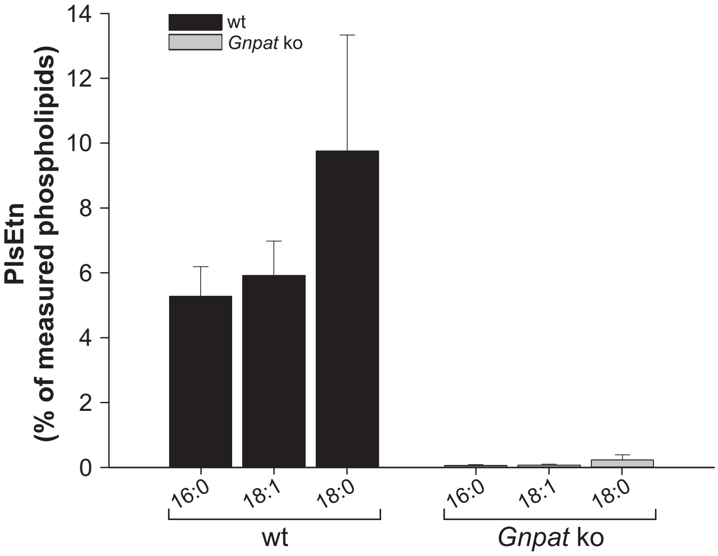

Supplementary Figure 3. ***C18:0 is the major sn-1 species in PlsEtn in the murine wild type brain gray matter.*** PlsEtn levels were determined in wild type (wt) and *Gnpat* knockout (ko) mouse gray matter brain tissue. Data are means ± SD of 10 (wt) and 4 (*Gnpat* ko) animals per group. 16:0, 18:0 and 18:1 indicate the different *sn-1* moieties.

Supplementary Table 1: Summary of detected phospholipid species

PlsEtn species	PC species	PE species	PS species	SM species
PlsEtn 16:0-16:0	PC 30:2	PE 32:2	PS 32:2	SM 16:1
PlsEtn 16:0-18:1	PC 30:1	PE 32:1	PS 32:1	SM 16:0
PlsEtn 18:1-16:0	PC 30:0	PE 34:2	PS 34:2	SM 18:1
PlsEtn 16:0-20:4	PC 32:3	PE 34:1	PS 34:1	SM 18:0
PlsEtn 16:0-20:3	PC 32:2	PE 36:5	PS 36:3	SM 20:1
PlsEtn 16:0-20:2	PC 32:1	PE 36:4	PS 36:2	SM 20:0
PlsEtn 18:1-18:1	PC 32:0	PE 36:3	PS 36:1	SM 22:1
PlsEtn 18:0-18:2	PC 34:4	PE 36:2	PS 36:0	SM 22:0
PlsEtn 18:0-18:1	PC 34:3	PE 36:1	PS 38:4	SM 24:2
PlsEtn 16:0-20:1	PC 34:2	PE 38:6	PS 38:3	SM 24:1
PlsEtn 16:0-22:6	PC 34:1	PE 38:5	PS 38:2	SM 24:0
PlsEtn 18:1-20:4	PC 34:0	PE 38:4	PS 38:1	
PlsEtn 16:0-22:5	PC 36:6	PE 38:3	PS 40:7	
PlsEtn 18:0-20:5	PC 36:5	PE 38:2	PS 40:6	
PlsEtn 16:0-22:4	PC 36:4	PE 38:1	PS 40:5	
PlsEtn 18:0-20:4	PC 36:3	PE 40:7	PS 40:4	
PlsEtn 18:1-20:3	PC 36:2	PE 40:6	PS 40:3	
PlsEtn 16:0-22:3	PC 36:1	PE 40:5		
PlsEtn 18:1-20:2	PC 36:0	PE 40:4		
PlsEtn 18:0-20:3	PC 38:6	PE 40:3		
PlsEtn 16:0-22:2	PC 38:5			
PlsEtn 18:1-20:1	PC 38:4			
PlsEtn 18:0-20:2	PC 38:3			
PlsEtn 18:0-20:1	PC 38:2			
PlsEtn 18:1-22:6	PC 38:1			
PlsEtn 18:0-22:6	PC 38:0			
PlsEtn 18:1-22:5	PC 40:6			
PlsEtn 18:0-22:5	PC 40:5			
PlsEtn 18:1-22:4	PC 40:4			
PlsEtn 18:0-22:4				
PlsEtn 18:1-22:3				
PlsEtn 18:1-22:2				
PlsEtn 18:0-22:3				
